# Vismodegib, an antagonist of hedgehog signaling, directly alters taste molecular signaling in taste buds

**DOI:** 10.1002/cam4.350

**Published:** 2014-10-30

**Authors:** Hyekyung Yang, Wei-na Cong, Jeong Seon Yoon, Josephine M Egan

**Affiliations:** 1 Laboratory of Clinical Investigation, National Institute on Aging, National Institutes of HealthBaltimore, Maryland; 2 Laboratory of Neuroscience, National Institute on Aging, National Institutes of HealthBaltimore, Maryland

**Keywords:** Basal-cell carcinoma, sonic hedgehog, taste buds, taste perception, Vismodegib

## Abstract

Vismodegib, a highly selective inhibitor of hedgehog (Hh) pathway, is an approved treatment for basal-cell carcinoma. Patients on treatment with vismodegib often report profound alterations in taste sensation. The cellular mechanisms underlying the alterations have not been studied. Sonic Hh (Shh) signaling is required for cell growth and differentiation. In taste buds, Shh is exclusively expressed in type IV taste cells, which are undifferentiated basal cells and the precursors of the three types of taste sensing cells. Thus, we investigated if vismodegib has an inhibitory effect on taste cell turnover because of its known effects on Hh signaling. We gavaged C57BL/6J male mice daily with either vehicle or 30 mg/kg vismodegib for 15 weeks. The gustatory behavior and immunohistochemical profile of taste cells were examined. Vismodegib-treated mice showed decreased growth rate and behavioral responsivity to sweet and bitter stimuli, compared to vehicle-treated mice. We found that vismodegib-treated mice had significant reductions in taste bud size and numbers of taste cells per taste bud. Additionally, vismodegib treatment resulted in decreased numbers of Ki67- and Shh-expressing cells in taste buds. The numbers of phospholipase C*β*2- and *α*-gustducin-expressing cells, which contain biochemical machinery for sweet and bitter sensing, were reduced in vismodegib-treated mice. Furthermore, vismodegib treatment resulted in reduction in numbers of T1R3, glucagon-like peptide-1, and glucagon-expressing cells, which are known to modulate sweet taste sensitivity. These results suggest that inhibition of Shh signaling by vismodegib treatment directly results in alteration of taste due to local effects in taste buds.

## Introduction

Basal-cell carcinoma (BCC) is the most common nonmelanoma skin cancer in the United States, with an annual incidence rate of approximately 1.5% that continues to increase 1. BCC is associated with mutations in components of the hedgehog (Hh) signaling pathway. Hh is a key regulator of cell growth and differentiation during development and controls epithelial and mesenchymal interactions in many tissues during embryogenesis. Hh binds to its receptor patched homologue 1 (PTCH1) and prevents PTCH1-mediated inhibition of signaling by smoothened homologue (SMO). Signaling by SMO results in the activation of transcription factors encoded by GLI family zinc finger (GLI) and consequent induction of Hh target genes, including GLI1 2. Basal-cell tumors have mutations in the Hh signaling pathway that inactivate PTCH1 (loss-of-function mutation) or, less commonly, constitutively activate SMO (gain-of function mutation) 3–5. These mutations cause constitutive activation of Hh signaling that may result in uncontrolled proliferation of basal cells. Thus, blocking the Hh pathway is a therapeutic option in patients with BCC 6,7.

Vismodegib (Erivedge®, Genentech-Curis) is the first oral medicine approved by the US Food and Drug Administration for the treatment of adults with advanced BCC (refer to both locally advanced and distantly metastatic BCCs) that has recurred after surgery or cannot be resected or irradiated. It selectively inhibits SMO, a central mediator of Hh signaling 8–12.

Intriguingly, patients on treatment with vismodegib often report alterations in taste sensation (dysgeusia) 9–11. In a trial conducted by Von Hoff et al., dysgeusia as a grade 2 adverse event occurred in six percent of patients 9. Sekulic et al. reported dysgeusia in 51% of recipients, which was mild to moderate in severity 10. Dysgeusia is also a class effect because it has been reported to occur with other SMO inhibitors, such as sonidegib 13–15: the therefore is likely to be mechanism-related 16–18. However, to date, there is no evidence for direct effects of systemic vismodegib administration in taste buds.

Taste cells are organized within onion-shaped taste buds that reside in three types of papillae in the tongue; fungiform, foliate, and circumvallate papillae. Fungiform and foliate papillae are present on the anterior two-thirds and the posterior sides of the tongue, respectively, while circumvallate papillae, containing the greatest numbers of taste buds, are located under the uvula 19. The taste cells contain the signaling molecules to detect all five prototypic types of taste: sweet, umami, bitter, salty, and sour 20. Taste cells are maintained by continuous cell renewal and the average taste cell lifespan is approximately 10–16 days 21,22. Taste cells are categorized into four types (types I–IV) 20. Type I cells have chemosensing (for salt taste) and supporting functions 23. Type II cells are primary chemosensing that contain the molecules for detecting sweet, umami, and bitter tastes: *α*-gustducin, T1 receptors (T1R) that detect sweet, including natural sweet tasting food, sweeteners and umami, and T2R that detect bitter 24,25. Type III cells have direct afferent contacts, considered to be the neuronal output cells, and they contain many neurotransmitters as well as the molecular machinery for detecting sour taste 26,27. Type IV cells are nonpolarized, undifferentiated cells located at the base of taste buds, in which sonic Hh (Shh) is exclusively expressed. They are the precursors of the remaining three types (types I–III) of taste cells in taste buds 28. Additionally, taste cells contain many hormones, cholecystokinin, glucagon, glucagon-like peptide-1 (GLP-1), vasoactive intestinal peptide, neuropeptide Y, and ghrelin, that modulate the perception of prototypic tastes 29–32.

In addition to its role in proliferation of type IV cells, Shh plays a critical role in development and patterning of taste papillae in rodents 33. The steroidal alkaloid, cyclopamine, which is a selective disruptor of Shh signaling pathway, or a Shh-blocking antibody altered fungiform papilla induction and distribution in embryonic rat tongue cultures 33. Here we hypothesized that vismodegib inhibits Shh signaling in taste cells, leading to disruption of taste cell turnover, that results, over time, in taste disturbance. We show that vismodegib treatment causes alteration in taste bud morphology and expression of taste sensing machinery in taste cells. Our results suggest that inhibition of Shh signaling by vismodegib treatment directly results in alteration of taste due to local effects in taste buds.

## Materials and Methods

### Animal and tissue processing

All animal care and experimental procedures followed U.S. National Institutes of Health guidelines and were approved by the Animal Care and Use Committee of the National Institute on Aging. Male C57BL/6J mice (Jackson Laboratory, Bar Harbor, ME) were administered by daily oral gavage of 30 mg/kg vismodegib (LC Laboratories, Woburn, MA) in 0.5% methylcellulose and 0.2% Tween 80 (MCT) 34. Animals of both vehicle and vismodegib groups were euthanized after 15 weeks of treatment using isoflurane overdose and tongues were collected from each animal. The length of the study was to allow for at least four taste cell turnovers. Tongues were fixed in 10% neutral-buffered formalin (Sigma-Aldrich, St Louis, MO) for 1 h and then cryoprotected with 20% sucrose in 0.1 mol/L phosphate buffer overnight at 4°C. Serial sections (8–10 *μ*m thickness) were cut through circumvallate papillae using a cryostat (HM 500M, MICRON, GmbH, Germany). In order to obtain a systematic appreciation without bias of the entire papillae, each papilla was sectioned and every 10th section was saved onto a slide. As taste buds are approximately 80–100 *μ*m in length, sampling every 10th section ensured that no two sections were from the same taste bud.

### Taste behavioral tests

Two-bottle taste test was carried out as described previously 35,36. All tastants were prepared with purified water from the National Institute on Aging animal facility and reagent-grade chemicals were presented to the animals at room temperature. Two different testing protocols were used: one for normally preferred stimulus (sucrose; Sigma-Aldrich) and one for normally avoided stimulus (denatonium benzoate, DB; Sigma-Aldrich). Preference was characterized by calculating the ratio of tastant intake to water intake over 24 h.

### RNA isolation and real-time PCR of taste buds

Real-time RT-PCR experiments were performed on total RNA isolated from taste buds of foliate papillae and nontaste epithelial tissue devoid of taste cells as described previously 36. The reverse-transcriptase reactions were performed using qScript cDNA SuperMix (Quanta Biosciences, Gaithersburg, MD). Reverse-transcribed cDNAs were amplified using PerfeCTa SYBR Green SuperMix, UNG (Quanta Biosciences). Primer sequences were described in Table1. The data were normalized to glyceraldehyde-3-phosphate dehydrogenase (GAPDH) mRNA.

**Table 1:** Sequence of primers for real-time PCR

Gene	Forward primer	Reverse primer	Amplicon size (bp)
Gli1	ATGAGTGTCTTGCTGGGGTCT	ATCTGCTTGGGGTTCCTTACC	84
GAPDH	AACTTTGGCATTGTGGAAGG	GGATGCAGGGATGATGTTCT	132

### Immunohistochemistry

Following antigen retrieval with 10 mmol/L sodium citrate buffer (pH 6.0) at 98°C for 20 min, immunofluorescence analyses were performed as described previously 37. Sources and dilutions of the applied primary antibodies are listed in Table2. 4′,6-diamidino-2-phenylindole (DAPI, 1:5000 dilution; Sigma-Aldrich) was used for nuclear staining. No fluorescent staining was observed in any sections when the primary antibodies were omitted.

**Table 2:** Primary antibodies used in immunofluorescence analyses

Antigen	Host	Vender	Dilution
Ki67	Rat	eBioscience, San Diego, CA	1:100
Shh	Rabbit	Santa-Cruz, Santa Cruz, CA	1:100
*α*-Gustducin	Rabbit	Santa-Cruz, Santa Cruz, CA	1:200
PLC*β*2	Rabbit	Santa-Cruz, Santa Cruz, CA	1:200
T1R3	Goat	Santa-Cruz, Santa Cruz, CA	1:100
GLP-1	Mouse	US biological, Swampscott, MA	1:100
Glucagon	Mouse	Sigma-Aldrich	1:200

### Quantification of immunoreactive taste cells

Mouse taste bud images were collected using an LSM-710 confocal microscope (Carl Zeiss MicroImaging, Thornwood, NY) in single planes. Approximately 100–120 taste buds per group were analyzed as described previously 31,36,37. Cells were scored as immunopositive only if a nuclear profile was present within the cell. The total number of cells in the section was determined by counting the number of DAPI-stained nuclei present in each taste bud. Finally, the percentage of immunoreactive taste cells was calculated by dividing the number of immunopositive taste cells by the total number of the taste cells in each taste bud. Both image capture and data analysis was performed by trained researchers who were blind to the experimental and control conditions.

### Quantification of taste bud size and taste cell numbers per taste bud

Taste bud sizes were calculated in accordance with our previous methods 37. In brief, the perimeters of the taste bud from every 10th section were outlined and the corresponding area was computed by the Zeiss LSM Image Browser software (Carl Zeiss MicroImaging). Simultaneously, 20 taste buds were randomly selected at different regions of each tongue to count the number of cells in a single taste bud, where one nucleus corresponded to one cell on the section.

### Statistical analyses

All data represent means ± SEM from at least three independent experimental replicates. Error bars on graphs represent the ±95% confidence interval. One-way analysis of variance (ANOVA) with the Bonferroni post-hoc test was performed by GraphPad Prism 6.0 (GraphPad Software, Inc., La Jolla, CA) as appropriate. *P *<* *0.05 was considered statistically significant throughout the study.

## Results

### Vismodegib attenuates body growth rate and taste responsivity

**Figure 1:**
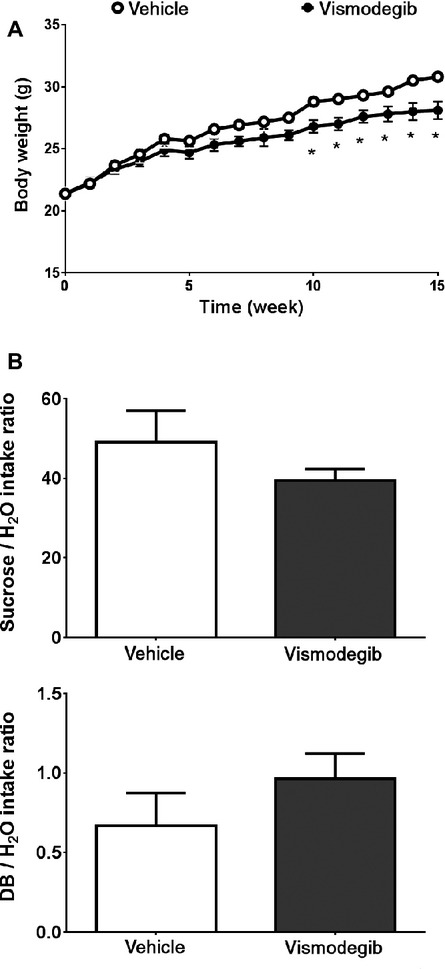
Effect of oral vismodegib and vehicle administration on body weight and hedgehog signaling in mice. (A) Growth curve for vehicle (open circle, *n *=* *8) and vismodegib (closed circle, *n *=* *8) during 15 weeks of treatment. Value are expressed as means ± SEM. *P <* *0.05 versus vehicle-treated group. (B) Two-bottle taste testing for modalities of sweet (600 mmol/L sucrose; top) and bitter taste (50 nmol/L denatonium benzoate, DB, bottom) in vehicle- and vismodegib-treated mice.

In patients received vismodegib for treatment of BCC, weight loss and taste disturbance were commonly reported 10,11. We monitored the body weight of vehicle- and vismodegib-treated mice during treatment for 15 weeks. After 4–5 weeks of treatment, the body weights of the two groups began to diverge and by 10 weeks of treatment, the weights of vismodegib-treated mice were significantly different from vehicle-treated mice (Fig.1A) and continued to diverge. By 15 weeks, vismodegib-treated mice were 9% lighter compared with vehicle-treated mice (*P *<* *0.05).

Next, we investigated potential differences in their taste perception. We tested the ability of both vehicle- and vismodegib-treated mice to detect sweet (sucrose) and bitter (DB, Fig.1B) by two-bottle taste test. We found that vismodegib-treated animals showed trends of reduced taste responsivity compared with vehicle-treated animals for sucrose and DB (Fig.1B; sucrose [top], *P *=* *0.371; DB [bottom], *P *=* *0.296).

### Vismodegib-GLI1 relationship

**Figure 2:**
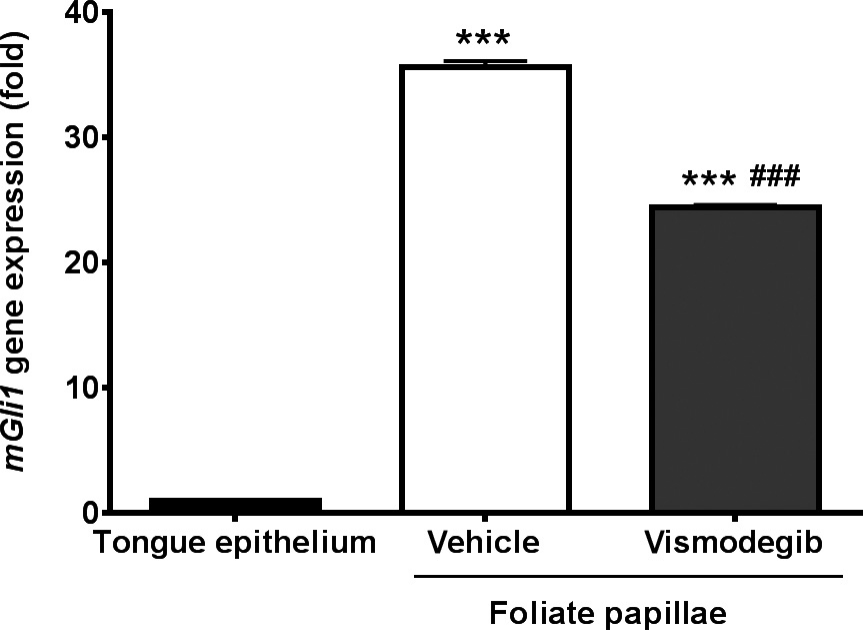
mRNA expression of the hedgehog target gene glioma-associated oncogene homolog 1 (*GLI*1) in a pool of taste buds from foliate papillae removed from tongues of vehicle- and vismodegib-treated mice. Tongue epithelium, devoid of taste buds, was used as a negative control. Values are expressed as means ± SEM. ***P <* *0.001 versus tongue epithelium, ^###^*P *<* *0.001 versus vehicle-treated group.

To determine whether vismodegib treatment suppresses Hh signaling in taste buds, we measured *Gli1* transcription, which is a downstream target and transcription factor of Hh signaling pathway, in foliate papillae from vehicle- and vismodegib-treated animals. As expected, vismodegib significantly decreased the Hh signaling, as shown by a 40% decrease in *Gli1* mRNA expression (Fig.2).

### Vismodegib alters taste bud size and number of taste cells per taste bud

**Figure 3:**
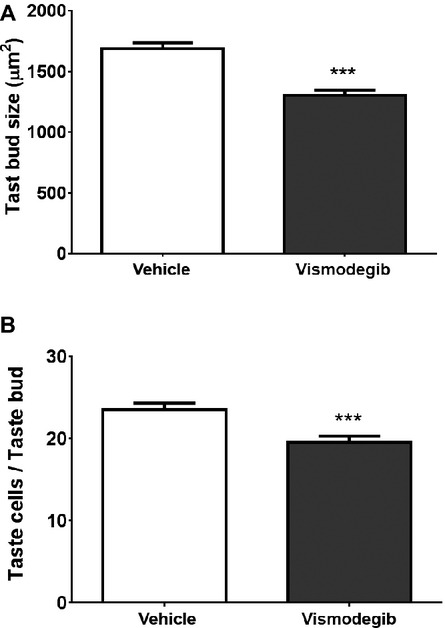
Taste bud size (A) and taste cell numbers per taste bud (B) in vehicle- and vismodegib-treated mice. Value are expressed as means ± SEM. ***P <* *0.001 versus vehicle-treated group.

To investigate whether the alteration in taste behavior and reduction in Hh signaling in taste buds from vismodegib-treated mice causes any alterations in taste bud morphology, we determined taste bud size and taste cell numbers per taste bud. We found that vismodegib-treated mice had significantly smaller taste buds (Fig.3A), compared to vehicle-treated mice and this was likely because there was a significant reduction in the numbers of taste cells within each taste bud in vismodegib-treated mice (Fig.3B).

### Vismodegib alters potential of taste cell renewal in taste bud

**Figure 4:**
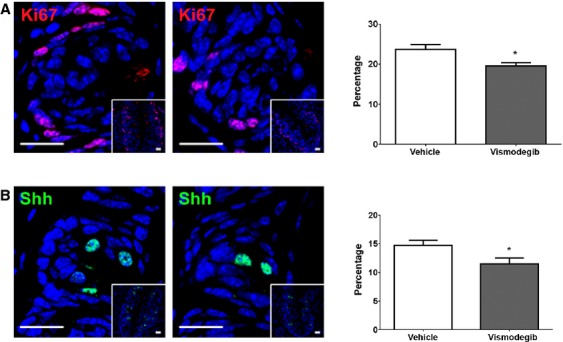
Expression of taste cell proliferation marker Ki67 (A) and Shh (B) in circumvallate papillae taste cells of vehicle- and vismodegib-treated mice. The histograms associated with each taste bud represent the percentage of immunoreactive taste cells containing each marker out of the total number of taste cells in each taste bud. All scale bars are 20 *μ*m. Blue is DAPI nuclear stain. Value are expressed as means ± SEM. *P <* *0.05 versus vehicle-treated group.

Adult taste buds are maintained by differentiation of type IV cells to any of the other three taste cell types 38,39. To investigate whether the alteration in taste bud size and taste cell numbers is associated with delayed cell renewal, we examined cell proliferation in taste buds by labeling with Ki67 as a marker of cell turnover. We found that vismodegib treatment resulted in decreased numbers of Ki67-positive cells in taste bud (Fig.4A). Additionally, there was a significant reduction in the number of Shh-expressing taste cells in vismodegib-treated animals compared with vehicle-treated animals (Fig.4B).

### Vismodegib alters taste sensing machinery and hormone expression in taste buds

**Figure 5:**
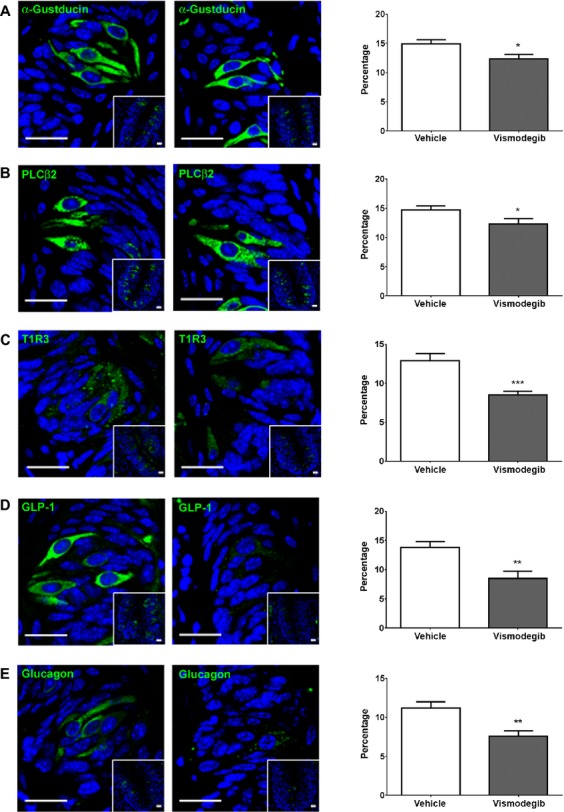
Expression of taste-modulating factors, *α*-gustducin (A), PLC*β*2 (B), T1R3 (C), GLP-1 (D), and glucagon (E), in circumvallate papillae taste cells of vehicle- and vismodegib-treated mice. The histograms associated with each taste bud represent the percentage of immunoreactive taste cells containing each marker out of the total number of taste cells in each taste bud. All scale bars are 20 *μ*m. Blue is DAPI nuclear stain. Value are expressed as means ± SEM. *P <* *0.05, **P <* *0.01, ***P <* *0.001 versus vehicle-treated group.

As we found alteration in taste modality and total taste cell number, we next investigated the expression of multiple taste-modulatory factors in the taste cells of vehicle- and vismodegib-treated mice. *α*-Gustducin and phospholipase C*β*2 (PLC*β*2) are essential chemosensing molecules in type II cells 24,40–42. *α*-Gustducin is the alpha subunit of the G-protein coupled to T1R and T2R and *α*-gustducin null mice have significantly reduced behavioral and/or nerve responses to bitter, sweet, and umami stimuli 40,43. PLC*β*2 is a key enzyme that is necessary for sweet-, umami-, and bitter-signal transduction. It is activated by *βγ* subunits of trimeric G proteins and it produces the 2-sec messengers, diacylglycerol, and inositol triphosphate, that connect taste receptor signals to downstream components of taste signal transduction ultimately to the brain 44. We found that the numbers of *α*-gustducin- and PLC*β*2-expressing cells were significantly reduced in vismodegib-treated animals compared with vehicle-treated mice (Fig.5A and B). T1R3 (the G protein-coupled receptor necessary for detecting sweet and umami) null mice have the greatly reduced gustatory nerve and behavioral responses to sugars and artificial sweeteners 45. We also found a significant reduction in the number of T1R3 immunoreactive cells in vismodegib-treated mice compared with vehicle-treated mice (Fig.5C). We previously reported that GLP-1 and glucagon are expressed in taste cells where they enhance sweet taste responsivity 30,46. As shown in Figure5D and E, there were reductions in the numbers of GLP-1- and glucagon-expressing cells as well as severely reduced GLP-1 and glucagon expression in vismodegib-treated animals compared with vehicle-treated animals.

## Discussion

In this study, we investigated the effects of vismodegib, given orally to mice so as to mimic how it is administered to patients, in order to provide a potential explanation for the distortion in taste perception that is reported to occur in up to 55% of patients taking vismodegib for BCC. In mice, oral vismodegib administration leads to decreased body weight, and reduction in the number of cells expressing sweet taste receptors and downstream signaling molecules. We also found a lower expression of hormones known to enhance sweet taste as well as a decrease in the numbers of cells expressing those hormones.

Taste cells have a limited lifespan, die by apoptosis and so must undergo replacement throughout life. Developmentally, they arise from Shh-expressing cells that have the properties of taste cell progenitors and that undergo continuous division 38,39. Either innervating nerves supply signals to the type IV cells to become specific ‘newborn’ taste cells or they are fate-committed cells that require signals from the nerves to become specifically terminally differentiated taste cells 47. These conclusions regarding neuronal input to taste bud maintenance derive from elegant experiments showing that denervation leads to disappearance of taste buds in the first few weeks after denervation followed by their reappearance once nerve regrowth has occurred [38]. It is also known that the type IV cells continuously undergo replication, as shown by Ki67, Shh and BrdU staining, so as to maintain sufficient precursor cell numbers continuously available to receive neuronal signals 38,39.

It appears that vismodegib causes a decrease in the numbers of type IV cells because of deceleration of their replication rate, as shown by a significant reduction in the number of cells positive for Ki67, a well-accepted marker of replicating cells, and Shh. And this, in turn, leads to a decrease in the numbers of cells capable of being in the ‘on’ mode so as to differentiate, on an as-needed basis, in order to refill the population of cells that undergoes death. The cells most affected by the reduction in Shh-positive numbers are the sweet taste receptor T1R3-expressing cells, in addition to GLP-1-and glucagon-containing cells; the receptor is necessary for sweet tasting and the hormones, both active fragments of the proglucagon molecule, locally enhance sweet taste responsivity.

Vismodegib is a competitive antagonist of SMO, a part of the Hh signaling pathway. Total SMO inhibition causes the transcription factor GLI1 to remain inactive, that in turn depresses expression of genes regulated by the Hh signaling pathway 2. In the present study *GLI1* expression in taste buds was decreased, but not fully inhibited, by vismodegib treatment in the mice. Even though not fully suppressed, this still resulted in a severe phenotype in taste cells. Therefore, complete suppression is not required to cause alterations in taste molecules.

To our knowledge, the current study is the first to demonstrate that vismodegib affects taste cell turnover and taste functional integrity. These findings provide an explanation for the changes in vismodegib-induced human taste perception because vismodegib most likely results in similar alterations in human taste buds.

## Conflict of Interest

None declared.
